# Reduced Effect of Anticonvulsants on AMPA Receptor Palmitoylation-Deficient Mice

**DOI:** 10.3389/fphar.2021.711737

**Published:** 2021-08-18

**Authors:** Madoka Iizumi, Akiko Oota-Ishigaki, Mariko Yamashita, Takashi Hayashi

**Affiliations:** ^1^ National Institute of Neuroscience, National Center of Neurology and Psychiatry (NCNP), Kodaira, Japan; ^2^ Biomedical Research Institute, National Institute of Advanced Industrial Science and Technology (AIST), Tsukuba, Japan

**Keywords:** phenytoin, trimethadione, anticonvulsant, palmitoylation, AMPA receptor, synapse, excitability

## Abstract

AMPA receptors are responsible for fast excitatory synaptic transmission in the mammalian brain. Post-translational protein *S*-palmitoylation of AMPA receptor subunits GluA1-4 reversibly regulates synaptic AMPA receptor expression, resulting in long-lasting changes in excitatory synaptic strengths. Our previous studies have shown that GluA1 C-terminal palmitoylation-deficient (GluA1C811S) mice exhibited hyperexcitability in the cerebrum and elevated seizure susceptibility without affecting brain structure or basal synaptic transmission. Moreover, some inhibitory GABAergic synapses-targeting anticonvulsants, such as valproic acid, phenobarbital, and diazepam, had less effect on these AMPA receptor palmitoylation-deficient mutant mice. This work explores pharmacological effect of voltage-gated ion channel-targeted anticonvulsants, phenytoin and trimethadione, on GluA1C811S mice. Similar to GABAergic synapses-targeting anticonvulsants, anticonvulsive effects were also reduced for both sodium channel- and calcium channel-blocking anticonvulsants, which suppress excess excitation. These data strongly suggest that the GluA1C811S mice generally underlie the excessive excitability in response to seizure-inducing stimulation. AMPA receptor palmitoylation site could be a novel target to develop unprecedented type of anticonvulsants and GluA1C811S mice are suitable as a model animal for broadly evaluating pharmacological effectiveness of antiepileptic drugs.

## Introduction

Various anticonvulsants are clinically used in the treatment of epileptic seizures (Beck and Elger, 2008; Goldenberg, 2010). Antiepileptic drugs (AEDs) affect several pharmacological targets to suppress excessive neuronal firing, resulting in prevention of the seizure spread in the brain. Disrupted excitatory/inhibitory (E/I) balance results in perturbed brain function and repetitive seizures leads to serious epilepsy (Paz and Huguenard, 2015). Despite the appropriate control of neuronal molecules such as ion channels and neurotransmitter receptors is crucial in the maintenance of the E/I balance (Haider and Mccormick, 2009; Isaacson and Scanziani, 2011; Xue et al., 2014), detailed mechanisms underlining epilepsy still remain unclear.

Over the past few decades, many seizure models in rodents have been established for the pharmacological testing of epileptic modifiers (Guillemain et al., 2012; Löscher, 2016; Löscher, 2017). Symptom-mimicking behavioral outcomes in these models provide an indicator of pharmacological effects on epileptic seizure. In addition to acute seizure models using drugs and chemically or electrically kindled rodents, genetically modified mice have been used to discover novel and highly effective anticonvulsants (Marshall et al., 2021). In these researches, many AEDs have been developed so far, which target ion channels, such as voltage-gated sodium channel (VGSC) α-subunits Na_V_1.1 and Na_V_1.2 and β-subunit (Kaplan et al., 2016), T-type voltage-gated calcium channel (VGCC) α1H-subunit Ca_V_3.2 (Zamponi et al., 2009), M-type potassium channel subunits (Springer et al., 2021), GABA_A_ receptor α1- and γ2-subunits (Treiman, 2001). These ion channels are implicated in some human epilepsy syndromes (Rogawski and Löscher, 2004). Targets ion channels are diverse and more specific anticonvulsants are in demand for more effective therapies with fewer side effects.

Glutamate is the major excitatory neurotransmitter in the mammalian central nervous system. Majority of the excitatory synapses throughout the mammalian brain contain α-amino-3-hydroxy-5-methyl-4-isoxazole propionate (AMPA)-type glutamate receptors, which are dominantly responsible for fast excitatory synaptic transmission. Therefore, basal excitatory synaptic transmission, synaptic plasticity, and higher brain function are broadly regulated by quantitative controls of postsynaptic AMPA receptor numbers (Collingridge et al., 2004; Shepherd and Huganir, 2007; Kessels and Malinow, 2009; Huganir and Nicoll, 2013). We have already shown that a key modification mechanism of AMPA receptor trafficking into and from excitatory synapses is the posttranslational protein *S*-palmitoylation of the receptors at their C-termini, the reversible attachment of the most abundant saturated fatty acid, palmitate (Hayashi et al., 2005; Jiang et al., 2006; Lin et al., 2009; Anggono and Huganir, 2012; Lu and Roche, 2012; Thomas and Hayashi, 2013; Lussier et al., 2015). Among the four AMPA receptor subunits GluA1-4 (also known as GluR1-4, GluRA-D or GluRα1-4), GluA1 mainly acts in activity-dependent AMPA receptor trafficking into postsynapses (Shepherd and Huganir, 2007; Heine et al., 2008; Anggono and Huganir, 2012).

Our previous studies have revealed that the GluA1 C-terminal palmitoylation regulates seizure susceptibility *in vivo* (Hayashi, 2021). GluA1 C-terminal palmitoylation-deficient (GluA1 Cys811 to Ser substituted, GluA1C811S) mice showed elevated seizure susceptibility and reduced anticonvulsive effects of AEDs (Itoh et al., 2018). Therefore, our seizure model mouse with single protein modification site-targeted mutation would be useful for effective evaluation of drugs. This may lead to development of unprecedented type of anticonvulsants. To confirm these possibilities, we further investigated pharmacological effects of a diverse group of anticonvulsants on GluA1C811S mice in this report. Our data exhibit that enhanced excitability based on deficiency of AMPA receptor palmitoylation bring less effect of broad AEDs.

## Materials and Methods

### Experimental Animals

For this study, we used previously generated GluA1C811S mutant mice (Itoh et al., 2018). The intercross of heterozygotes resulted in the production of wild-type, heterozygous, and homozygous offspring at the expected 1:2:1 Mendelian ratio. Wild-type and homozygous adult male mice were used for subsequent analyses. C57BL/6N mice were obtained from Charles River Laboratories Japan. Mice were fed with standard laboratory chow and water in standard animal cages under a 12 h light/dark cycle. Mice were used at the age of 3 months for phenytoin (PHT) and trimethadione (TMZ) experiments and 7 weeks for 2,3-dioxo-6-nitro-7-sulfamoyl-benzo[f]quinoxaline (NBQX) experiment.

All animal care and experiments were performed in accordance with the regulations and institutional guidelines of the National Center of Neurology and Psychiatry (NCNP), Japanese Pharmacological Society, and Japan Neuroscience Society. The technical protocols for animal experiments in this study were approved by the Institutional Review Committees of the National Institute of Neuroscience, NCNP.

### Drugs

Pentylenetetrazole (PTZ), PHT, TMZ and NBQX were purchased from Sigma-Aldrich. PHT, 20 mg/kg i.p., was initially dissolved by means of NaOH in distilled water (Klein et al., 2015). Briefly, PHT was dissolved in 75 mM NaOH as a stock solution (50 mg/ml) and then diluted 25 times in *phosphate-buffered saline* (PBS) for i.p. injection. PTZ, 60 mg/kg i.p., TMZ, 300 mg/kg i.p., and NBQX, 30, 60 or 100 mg/kg i.p., were directly dissolved in PBS. All reagents were prepared at time of use.

### Seizure Observation and Scoring

PTZ was administered intraperitoneally at a dose of 60 mg/kg (Itoh et al., 2018). Control mice were injected with PBS. PHT, TMZ or NBQX was administered intraperitoneally for indicated periods before PTZ injection. We examined seizure events during the 20 min observation period after injection. Seizure intensity was scored as follows: stage 0, no response; stage 1, ear, mouth and facial twitching; stage 2, head nodding and convulsive twitching axially through the body; stage 3, limbic myoclonus; stage 4, rearing demonstrated by standing on hind legs and wild jumping; stage 5, generalized tonic-clonic seizures; and stage 6, death (Racine score). The highest seizure scores during the observation period were used for evaluation.

### Statistical Analysis

Statistical analyses were performed using Prism 8 (GraphPad Software) and Excel (Microsoft). Two-way ANOVA followed by Fisher’s LSD test was used to compare two groups. All data are expressed as mean ± standard error of the mean (s.e.m.).

## Results

### Reduced Effects of a Sodium Channel-Targeting Anticonvulsant, Phenytoin, in GluA1C811S Mutant Mice

Our previous studies have revealed that palmitoylation at GluA1 Cys811 suppress hyperexcitation *in vivo* (Itoh et al., 2018). Compared with wild-type mice, elevated seizure susceptibility was observed in palmitoylation-deficient GluA1C811S mice, when they are administrated with a non-competitive GABA_A_ receptor antagonist, pentylenetetrazole (PTZ). Disturbance of inhibitory GABAergic synapses leads to instability of neuronal network in the brain (Treiman, 2001). Although both wild-type and GluA1C811S mice showed dose dependent effects of PTZ on inhibitory synapse suppression-induced seizure, GluA1C811S mutant mice exhibited severer clonic and/or tonic convulsion, jumping, and wild running (classified in stage 4 or 5, Racine score) during 20 min observation after intraperitoneal PTZ injection at a dose of 60 mg/kg. Our experimental data also showed reduced effects of inhibitory GABAergic synapses-targeting anticonvulsants, valproic acid (VPA), phenobarbital (PB), and diazepam (DZP) on PTZ-induced seizure in GluA1C811S mice.

**FIGURE 1:**
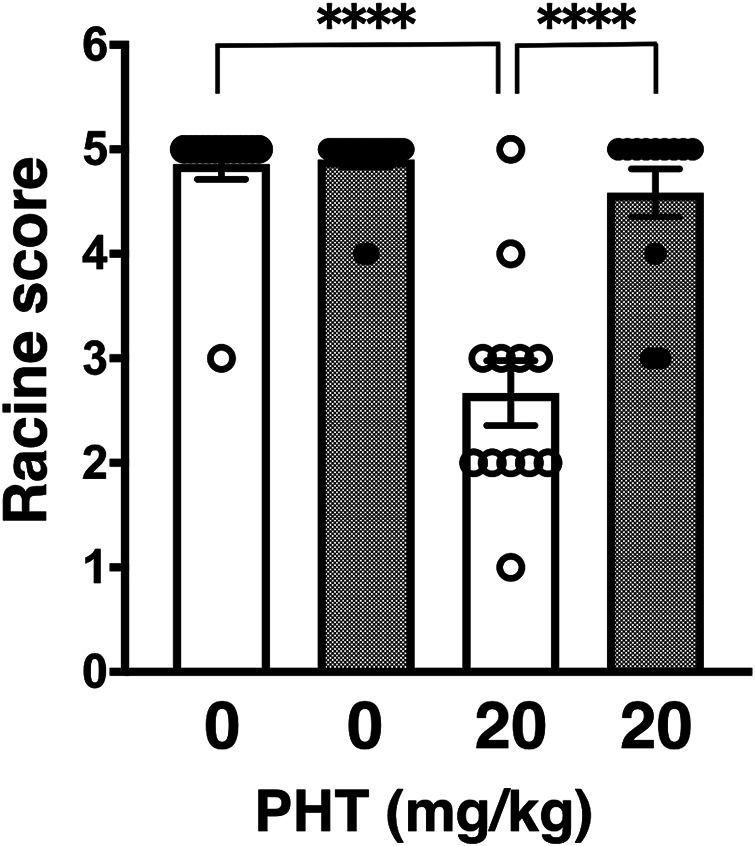
Inhibitory effects of phenytoin (PHT) on PTZ-evoked seizure. Effects of PHT on occurrence of generalized tonic-clonic seizures. Seizure scores of adult male wild-type (white dots and white bars) and GluA1C811S (black dots and hashed bars) mice received an administration of PHT at a dose of 0 or 20 mg/kg, 30 min before a single injection of 60 mg/kg PTZ. Four bars: *n* = 14, 20, 12, 12 mice (left to right). Error bars represent s.e.m. ANOVA with Fisher’s LSD *post hoc* test. ****p < 0.0001.

In this study, we proceeded to test other anti-epileptic agents, which have different pharmacological points of action. First, we examined phenytoin (PHT), a hydantoin derivative, which acts as an anticonvulsant by blocking VGSCs. PHT administered intraperitoneally at an effective dose of 20 mg/kg significantly suppress PTZ-induced seizures in wild-type mice (Figure 1: 4.86 ± 0.14, *n* = 14 for PBS-injected wild-type mice; 2.70 ± 0.33, *n* = 12 for PHT-injected wild-type mice) as previously reported (Klein et al., 2015; Song et al., 2018). On the other hand, PHT showed reduced anticonvulsive effects on PTZ-injected GluA1C811S mice (Figure 1: 4.90 ± 0.07, *n* = 20 for PBS-injected GluA1C811S mice; 4.58 ± 0.23, *n* = 12 for PHT-injected GluA1C811S mice, Two-way ANOVA, genotype × treatment interaction *F*_1, 54_ = 26.22, *p* < 0.0001). There was no obvious difference between PBS-injected wild-type and GluA1C811S mice (*p* = 0.8575). Abnormal freezing behaviors were observed just by injecting PHT at higher dose (50 mg/kg for 30 min) without PTZ administration (data not shown).

### Reduced Effects of a Calcium Channel-Targeting Anticonvulsant, Trimethadione, in GluA1C811S Mutant Mice

**FIGURE 2:**
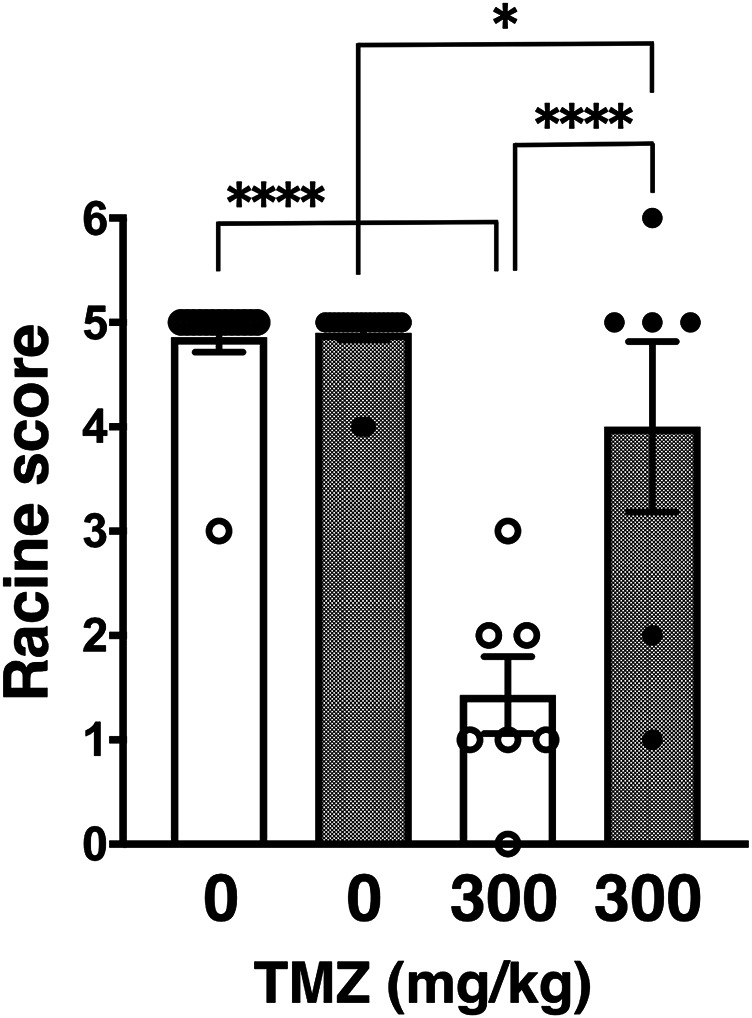
Inhibitory effects of trimethadione (TMZ) on PTZ-evoked seizure. Effects of TMZ on occurrence of generalized tonic-clonic seizures. Seizure scores of adult male wild-type (white dots and white bars) and GluA1C811S (black dots and hashed bars) mice received an administration of TMZ at a dose of 0 or 300 mg/kg, 30 min before a single injection of 60 mg/kg PTZ. Four bars: *n* = 14, 20, 7, 6 mice (left to right). Error bars represent s.e.m. ANOVA with Fisher’s LSD *post hoc* test. *p < 0.05, ****p < 0.0001.

Next, we tested trimethadione (TMZ), an oxazolidinedione derivative, which possesses anti-seizure activity by inhibiting T-type VGCCs (Zamponi et al., 2009). Similar to other examined anticonvulsants in a series of our studies, TMZ also showed anticonvulsive effect on PTZ-induced seizures in wild-type and GluA1C811S mice at a dose of 300 mg/kg (Barton et al., 2005; Pastore et al., 2014). However, TMZ showed less effect on PTZ-induced seizures in GluA1C811S mice at an effective dose of 300 mg/kg (Figure 2: 4.90 ± 0.07, *n* = 20 for PBS-injected GluA1C811S mice; 4.00 ± 0.82, *n* = 6 for TMZ-injected GluA1C811S mice), compared with wild-type mice (Barton et al., 2005; Pastore et al., 2014) (Figure 2: 4.86 ± 0.14, *n* = 14 for PBS-injected wild-type mice; 1.43 ± 0.37, *n* = 7 for TMZ-injected wild-type mice, Two-way ANOVA, genotype × treatment interaction *F*_1, 43_ = 20.43, *p* < 0.0001.).

### Anticonvulsive Effects of a AMPA Receptor Blocker, NBQX, in GluA1C811S Mutant Mice

**FIGURE 3:**
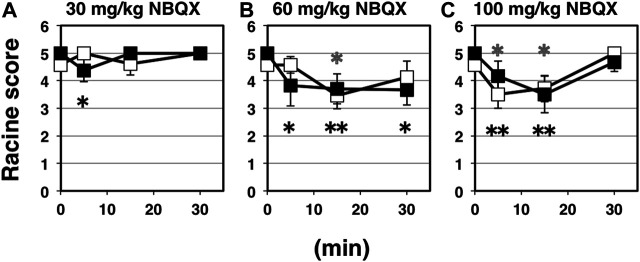
Inhibitory effects of NBQX on PTZ-evoked seizure. **(A)** Time course of NBQX effects at doses of 30 mg/kg. Seizure scores of 7 weeks old male wild-type (white squares) and GluA1C811S (black squares) mice received an administration of NBQX at a dose of 0 or 30 mg/kg for indicated periods before a single injection of 60 mg/kg PTZ. Four white squares (wt): *n* = 19, 6, 5, 4 mice, four black squares [GluA1C811S]: n = 19, 8, 4, 4 mice (left to right). **(B)** Time course of NBQX effects at doses of 60 mg/kg. Seizure scores of 7 weeks old male wild-type (white squares) and GluA1C811S (black squares) mice received an administration of NBQX at a dose of 0 or 60 mg/kg for indicated periods before a single injection of 60 mg/kg PTZ. Four white squares (wt): *n* = 19, 7, 11, 8 mice, four black squares [GluA1C811S]: *n* = 19, 6, 10, 9 mice (left to right). **(C)** Time course of NBQX effects at doses of 100 mg/kg. Seizure scores of 7 weeks old male wild-type (white squares) and GluA1C811S (black squares) mice received an administration of NBQX at a dose of 0 or 100 mg/kg for indicated periods before a single injection of 60 mg/kg PTZ. Four white squares (wt): *n* = 19, 6, 7, 3 mice, four black squares [GluA1C811S]: *n* = 19, 6, 6, 3 mice (left to right). Error bars represent s.e.m. ANOVA with Fisher’s LSD *post hoc* test. *p < 0.05, **p < 0.01, compared to PBS controls. Colorless asterisks indicate wild-type and black asterisks indicate GluA1C811S mice.

NBQX is a highly selective competitive antagonist of AMPA- and kainite-type glutamate receptors, which is widely used because of its well-established anticonvulsant activity in rodent seizure models. The vast majority of fast excitatory synaptic transmission is mediated by AMPA receptors (Collingridge et al., 2004; Shepherd and Huganir, 2007; Kessels and Malinow, 2009; Huganir and Nicoll, 2013). Basically, NBQX is expected to inhibit synaptic AMPA receptor activities, whether AMPA receptors are palmitoylated or not. Namely, NBQX suppress excitatory currents through surface-expressing AMPA receptors on postsynapses, which should consist of both palmitoylated and depalmitoylated receptors (Nakagawa, 2010). Intraperitoneal application of NBQX as a representative of glutamatergic excitatory synapse-targeting anticonvulsant for indicated periods brought significant anticonvulsive effects on both wild-type and GluA1C811S mice (Figure 3A: 30 mg/kg NBQX; Figure 3B: 60 mg/kg NBQX; Figure 3C: 100 mg/kg NBQX). While anticonvulsive effects were hardly observed at a low dose (Figure 3A: 4.58 ± 0.21, *n* = 19 for 0 min, 5.00 ± 0.00, *n* = 6 for 5 min, 4.60 ± 0.40, *n* = 5 for 15 min, 5.00 ± 0.00, *n* = 4 for 30 min, PBS-injected wild-type mice; 5.00 ± 0.00, *n* = 19 for 0 min, 4.38 ± 0.42, *n* = 8 for 5 min, 5.00 ± 0.00, *n* = 4 for 15 min, 5.00 ± 0.00, *n* = 4 for 30 min, NBQX-injected GluA1C811S mice, Two-way ANOVA, effect of treatment *F*_3, 61_ = 0.364, *p* = 0.7792), higher concentration of NBQX suppresses PTZ-induced seizure (Figure 3B: 4.58 ± 0.21, *n* = 19 for 0 min, 4.57 ± 0.30, *n* = 7 for 5 min, 3.45 ± 0.47, *n* = 11 for 15 min, 4.13 ± 0.58, *n* = 8 for 30 min, PBS-injected wild-type mice; 5.00 ± 0.00, *n* = 19 for 0 min, 3.83 ± 0.75, *n* = 6 for 5 min, 3.70 ± 0.54, *n* = 10 for 15 min, 3.67 ± 0.55, *n* = 9 for 30 min, NBQX-injected GluA1C811S mice, Two-way ANOVA, effect of treatment *F*_3, 81_ = 4.829, *p* = 0.0038; Figure 3C: 4.58 ± 0.21, *n* = 19 for 0 min, 3.50 ± 0.50, *n* = 6 for 5 min, 3.71 ± 0.47, *n* = 7 for 15 min, 5.00 ± 0.00, *n* = 3 for 30 min, PBS-injected wild-type mice; 5.00 ± 0.00, *n* = 19 for 0 min, 4.17 ± 0.54, *n* = 6 for 5 min, 3.50 ± 0.67, *n* = 6 for 15 min, 4.67 ± 0.33, *n* = 3 for 30 min, NBQX-injected GluA1C811S mice, Two-way ANOVA, effect of treatment *F*_3, 63_ = 6.941, *p* = 0.0004). Our data showed that there was no significant difference between palmitoylatable wild-type and non-palmitoylated GluA1C811S mice at each time point (Figure 3A: Two-way ANOVA, genotype × treatment interaction *F*_3, 61_ = 2.157, *p* = 0.1023; Figure 3B: Two-way ANOVA, genotype × treatment interaction *F*_3, 81_ = 0.9806, *p* = 0.4061; Figure 3C: Two-way ANOVA, genotype × treatment interaction *F*_3, 63_ = 0.5016, *p* = 0.6825). PTZ-induced seizures cannot be fully suppressed by NBQX and mice treated with 100 mg/kg NBQX for 30 min seem to exhibit less anticonvulsive effect at a glance. These are because some NBQX-treated mice fell down or stayed immobile in a low position after standing up on hind legs in our observation. According to the Racine score, stage 4 or 5 was recorded in these cases. These abnormal physical reactions induced by application of NBQX at high concentration for long periods may cause the poor numerically outcome for NBQX anticonvulsive score at the condition.

## Discussion

Epilepsy is characterized by unpredictable, unprovoked recurrent seizures, resulting from the abnormally synchronous activity of excitatory neurons in a focal area of the cerebrum. The sudden rush of electrical activity may convey throughout the entire brain in some cases. Epilepsy can last from undetectable short periods to long periods that appears sporadically and continuously evoke vigorous shaking (Kwan and Brodie, 2000; Grone and Baraban, 2015; Staley, 2015). Epileptic seizures are associated with excessive cortical excitability resulting from imbalances between excitation and inhibition in some focal regions of the cerebrum. Disturbance of neuronal network stability induce many types of epileptic seizure and various medicines are selected, depending on the symptom.

Besides brain tumors and traumatic brain injuries, genetic dysfunctions are the predominant determinants for epilepsy (Guillemain et al., 2012; Löscher, 2016; Löscher, 2017; Marshall et al., 2021). More than a hundred genes have been identified as causative genes for epilepsy to date and a lot of epilepsy-related mutations locate on loci coding ion channels (Helbig and Ellis, 2020). Accumulated efforts aimed at improved therapies have revealed many monogenic and combination of factors. As it has been studied for a long time, major excitatory neurotransmission is mediated by glutamate in the mammalian brain. Especially, AMPA receptor palmitoylation-dependent regulation of glutamatergic synapses is a *prevailing* mechanism to control the excitatory synaptic strength in the maintenance of network stability (Hayashi, 2021). In addition to our previous verification using AMPA receptor palmitoylation-deficient (GluA1C811S) mice with GABAergic synapses-targeting anticonvulsants, we further showed in this report that reduced anticonvulsive effects are observed when GluA1C811S mice are treated by voltage-gated ion channel-blocking anticonvulsants, PHT (Figure 1) and TMZ (Figure 2). Difference of anticonvulsive effect between PHT and TMZ may depend on each target. VGSCs and T-type VGCCs show non-overlapping distribution in the complex cerebral network, in which glutamatergic synaptic transmissions occupy most there (Zamponi et al., 2009; Kaplan et al., 2016; Wang et al., 2017). VGSCs are the basic ion channels for neuronal excitability, which are crucial for the resting potential and the generation and propagation of action potentials in neurons (Wang et al., 2017). As clinical treatment, PHT is useful for the prevention of tonic-clonic seizures and focal seizures, but not absence seizures in human (Kaplan et al., 2016). On the other hand, TMZ is most commonly used to treat epileptic conditions that are resistant to other treatments. When excitatory AMPA receptors themselves are inhibited by pre-injected NBQX, which ubiquitously suppress excitation of glutamatergic synapses in the whole brain, similar level of anticonvulsive effects was observed in both wild-type and palmitoylation-deficient GluA1C811S mice (Figure 3). This means that both palmitoylated and depalmitoylated AMPA receptors were inhibited by NBQX on excitatory synapses in the brain.

These experimental data presented in this report indicate that single protein modification site such as AMPA receptor palmitoylated cysteine become a novel target to develop unprecedented type of anticonvulsants. Moreover, our GluA1C811S mouse could be useful as a general animal model to evaluate newly developed AED candidates, by comparing with current drugs. Anticonvulsants are also increasingly being used in the treatment of mental disorders and neuropathic pain (Keck et al., 1998; Rogawski and Löscher, 2004; Joshi et al., 2019). Abnormalities are associated with accumulating brain damage and neurological dificits. Palmitoylation-deficient GluA1C811S mice may be widely applicable to test drugs to treat brain disorders in the future.

## Data Availability

The original contributions presented in the study are included in the article/supplementary material, further inquiries can be directed to the corresponding author.
